# CannabinEYEds: The Endocannabinoid System as a Regulator of the Ocular Surface Nociception, Inflammatory Response, Neovascularization and Wound Healing

**DOI:** 10.3390/jcm9124036

**Published:** 2020-12-14

**Authors:** Francesco Aiello, Gabriele Gallo Afflitto, Ji-Peng Olivia Li, Alessio Martucci, Massimo Cesareo, Carlo Nucci

**Affiliations:** 1 Ophthalmology Unit, Department of Experimental Medicine, University of Rome “Tor Vergata”, 00133 Rome, Italy; gabgallo9@gmail.com (G.G.A.); alessio.martucci@live.it (A.M.); massimo.cesareo@uniroma2.it (M.C.); nucci@med.uniroma2.it (C.N.); 2 Moorfields Eye Hospital NHS Foundation Trust, London EC1V 2PD, UK; olivialieyes@gmail.com

**Keywords:** endocannabinoid system, CB1-receptor, CB2-receptor, TRPV1, PPAR, ECS, ocular surface, conjunctiva, cornea, dry eye

## Abstract

The endocannabinoid system (ECS) is a complex regulatory system, highly conserved among vertebrates. It has been widely described in nearly all human tissues. In the conjunctiva and cornea, the ECS is believed to play a pivotal role in the modulation of the local inflammatory state as well as in the regulation of tissue repair and fibrosis, neo-angiogenesis and pain perception. This review aims to summarize all the available data on ECS expression and its function in ocular surface structures to provide a specific insight concerning its modulation in dry eye disease, and to propose directions for future research.

## 1. Introduction

Cornea and conjunctiva together constitutes the ocular surface of the eye [1]. They play important functional roles, being responsible for ocular mechanical and immunological protection, among other functions [2]. Their homeostasis relies on a finely-regulated network of cellular (i.e., corneal epithelial cells, keratocytes, and immune cells) and soluble components (i.e., cytokines, chemokines, and growth factors). Together, all these elements are responsible for the maintenance of epithelial integrity, the promotion of a balanced inflammatory response and the prevention of aberrant wound healing process [2].

Endocannabinoids (ECB) are endogenous lipid mediators able to bind to and to activate cannabinoid (CB) and non-cannabinoid receptors (e.g., transient receptor potential cation channel subfamily V member 1 (TRPV1) and peroxisome proliferator-activated receptors (PPARs)), which constitute the primary molecular targets responsible for the biological effects of the Δ^9^-tetrahydrocannabinol (Δ^9^-THC) [3,4]. The ECB system (ECS) has been widely described in nearly all human tissues. Among other functions, the ECS acts to regulate inflammation and immune response. For instance, both 2-arachidonoylglycerol (2-AG) and *N*-arachidonoylethanolamine or anandamide have been shown to inhibit cyclooxygenase activity [5,6]. In addition, endocannabinoids were found to prevent NFκB activation and its downstream pro-inflammatory cascade, via direct inhibition on I-kB kinase [7,8]. Nonetheless, anandamide (AEA) demonstrated the ability to reduce mitogen-induced T- and B-lymphocyte proliferation, probably because of increased apoptosis [9]. In addition, as shown in a number of studies, both 2-AG and anandamide are able to induce nitric oxide release through binding to their specific receptor [10,11,12].

Even in conjunctiva and cornea, the ECS is thought to play a pivotal role in the modulation of the local inflammatory state as well as in the regulation of tissue repair and fibrosis, neo-angiogenesis and pain perception [13].

This review aims to summarize all the available data regarding the expression and function of some of the key player of the ECS in the ocular surface structures and to provide a specific insight concerning its modulation in dry eye disease. Future directions are proposed to highlight the specific areas which should be prioritized for research.

## 2. Materials and Methods

A literature review using the PubMed database over the past 10 years was performed using combinations and variations of the following terms: Ocular surface, conjunctiva, cornea, tear film, cannabinoid, endocannabinoid, CB1r, CB2r, PPAR, TRPV, anandamide, AEA, 2-arachidonoylglycerol, 2-AG, dry eye, dry eye disease, DED, cannabidiol, neoangiogenesis, fibrosis, wound healing, immune infiltration, inflammation, nitric oxide and prostaglandin. A total of 357 articles were found. The following records were excluded: Duplicates and non-English reports. Studies assessing non-ocular surface pathology were also excluded.

## 3. ECS in the Ocular Surface: An Overview

The ECS is a complex regulatory system, highly conserved among vertebrates. In more recent years, it has been shown to be responsible for the regulation of several physiological functions, including immune response and pain perception as well as metabolic, neuronal and cardiovascular activities [14,15]. The characterization of the ECS stemmed from the isolation of the Δ^9^-THC from cannabis in 1964. Afterwards, the CB1 receptor (CB1r) was discovered and cloned in 1990, followed by the identification of endogenous ligands [4,16].

## 4. Cannabinoid Receptors

The two main receptors of the ECS are the CB1r and CB2 receptor (CB2r) [17,18]. These are G-proteins coupled receptors (GPCRs) able to both detect molecules outside the cell and interact with other G proteins to activate internal signal transduction pathways.

CB1r is the most highly expressed GPCR in the central nervous system, especially in the cerebral cortex, nuclei basales, hippocampus and cerebellum, and it is detectable in peripheral nerve endings, too [19,20]. CB2r is mainly expressed in the peripheral organs, tissues and cells of the immune system, such as tonsils, spleen, thymus, macrophages, T and B cells, natural killer cells, monocytes and neutrophils to a lesser extent [21,22].

Both in conjunctiva and cornea, CB1r and CB2r expression has been widely investigated. In 1999, Straiker et al. observed CB1r to be expressed on both corneal epithelium and endothelium, using post-mortem immunostained human corneas. However, no clear staining for CB1r was detected in the corneal stroma [23]. Subsequently, Iribarne et al. found the presence of CB1r and CB2r immunoreactivity in the epithelial layer of the human conjunctiva. CB2r expression was also observed in conjunctival vascular endothelium and stromal cells [24].

Similarly, Assimakopoulou et al. described an intense immunostaining for CB1r and CB2r in all tissue layers of human pterygium, in a case series including 32 eyes of 32 patients. In particular, they described a strong and moderate granular cytoplasmic immunoreactivity for CB1r and CB2r in the basal and suprabasal layers, respectively [25].

The functional role of CB1r and CB2r in response to induced corneal injury was evaluated by Suburo et al. [26]. They found that, after the application of a paper soaked in 20% ethanol on mice’s central cornea, both CB1r and CB2r immunoreactivity increased not only in the central cornea but also in the limbus, when compared to baseline. Furthermore, small CB2r expressing cells appeared in the corneal stroma. This data suggested the infiltration of immune cells into the injured tissue. As for the peculiar limbal immunoreactivity both in control and in injured corneas, CB1r and CB2r have been proposed by the authors as markers for limbal stem cells and transient amplifying cells. The increase in the immunostaining intensity and in the number of epithelial CB2r expressing cells following injury suggests that these receptors might be involved in the regulation of cell proliferation and differentiation [26].

CB1r and CB2r involvement in corneal healing response was also investigated by Murataeva et al., both in an in vitro and in an in vivo model [27,28]. They demonstrated not only an upregulation of the CB2r signaling in the injured mouse cornea but also found that both *CB1r* and *CB2r* gene deletion is responsible for the wound closure delay. Interestingly, they also proposed that both CB1r and CB2r activation is responsible for the regulation of the chemotactic process needed for wound healing. This evidence was suggested by a transient increase in the ocular surface levels of nearly all *N*-acyl-ethanolamines, including the CB1r/CB2r endogenous ligand anandamide, promoting these lipids as chemotactic factors [27,28].

Notably, Pisanti et al. demonstrated that the role of CB receptor activation during the course of a wound healing response even regulates the induction of neoangiogenic process [29]. As a proof of concept, they showed that the use of CB1r antagonists was able to inhibit fibroblast growth factor (FGF) induced neovascular sprouting in an in vivo rabbit model, without any effect on the pre-existing mature capillaries [29].

**Figure 1:**
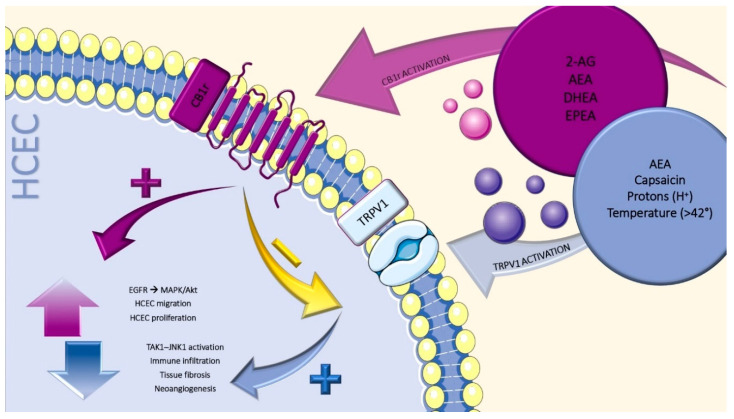
Agonism on cannabinoid receptor 1 (CB1r) by 2-arachidonoylglycerol (2-AG), *N*-arachidonoylethanolamine (AEA), docosahexaenoyl ethanolamide (DHEA) and eicosapentaenoyl ethanolamide (EPEA) is able to desensitize transient receptor potential cation channel subfamily V member 1 (TRPV1) (yellow arrow) and, via the epidermal growth factor receptor (EGFR)/MAPK, to promote human corneal epithelial cells (HCEC) migration and proliferation (violet pathway). The activation of transient receptor potential vanilloid-1 (TRPV1) elicit TAK1-JNK1 signaling cascade, which in turn is responsible for the promotion of tissue immune infiltration, fibrosis and neovascularization (blue pathway).

Apart from the aforementioned results, CB1r agonism was shown to be effective even in the modulation of capsaicin-evoked corneal pain responses and inflammation. In a mouse model of silver nitrate-induced corneal injury, Thapa et al. demonstrated that the use of positive modulators of CB1r was able to reduce both the pain score and the neutrophilic infiltration [30]. The same group had previously reported that activation of both CB1r and CB2r might reduce both the TRPV1-induced corneal pain response provoked via a capsaicin challenge and the neutrophilic corneal infiltration (Figure 1). These findings promoted the existence of a strict cross-talk between cannabinoid and non-cannabinoid receptors [31].

The aforementioned results strongly suggest that the role of the corneal CB receptors activation not only mediate the mere perception of nociceptive stimuli, but also co-operate in the activation of a cascade of events responsible for the re-establishment of local homeostasis. The findings from Bereiter et al. demonstrated to be highly consistent with this last hypothesis [32]. In fact, they showed that CB1r affects the activity of corneal-responsive neurons which contribute to homeostasis of the anterior segment of the eye rather than to the sensory-discriminative aspects of corneal nociception [32].

Given the plethora of related involvement, both CB1r and CB2r emerge as not mere regulators of nociception but as fine modulators of the corneal inflammatory state.

## 5. Non-Cannabinoid Receptors

Apart from CB1r and CB2r, a number of different membrane receptor families has been proved to be activated by endocannabinoid mediators and by their congeners. Among them, PPARs and TRPVs are worth mentioning for the biological activity expressed in the regulation of the ocular surface homeostasis.

## 6. Transient Receptors Potential Vanilloid-1

TRPV1 is a nonselective cationic-permeable channel, activated by capsaicin, protons, toxins and temperature in the noxious range (>42 °C), making it physiologically important for thermal and chemical nociception [33]. TRPV1 also interacts with the CB1r agonist, anandamide, both directly and through a specific cross-talk [34,35].

Functional TRPV1 channels may be found throughout the human body, including in the peripheral nerve endings, brain and spinal cord [36]. Additionally, TRPV1 expression has been constitutively detected on the ophthalmic branch of trigeminal nerve endings, in the corneal epithelial and endothelial cells and in the corneal stromal fibroblast [36,37].

The physiological importance of ocular surface TRPV1 expression has been widely demonstrated [36].

On sensory nerves and other cell types (i.e., epithelial and mesenchymal cell types) TRPV1 activation determines the release of tachykinin neuropeptides, such as substance P and calcitonin gene-related peptide [38].

In human corneal epithelial cells (HCEC), it has been shown that TRPV1 transactivation is able to elicit epidermal growth factor receptor (EGFR) signaling cascades [39]. As a result, a global MAPK and Akt/PI-3K pathway stimulation is induced [39]. These events were found to determine up to 3.3- and 9-fold increases in interleukins (IL)-6 and -8 release, respectively, through TAK1 activation of MAPK/JNK1-dependent and MAPK/JNK1-independent signaling pathways [40].

Moreover, TRPV1 signal supports transforming growth factor β1 (TGFβ1)-mediated myofibroblast transdifferentiation of stromal keratocytes, a mechanism considered to be responsible for corneal opacification [36]. However, even though TRPV1 activation promotes corneal tissue repair following an incision injury, a more severe corneal alkali-burn results in persisting inflammation and tissue fibrosis in mice, with a consequent, unfavorable visual outcome [41,42]. Similarly, it has been observed that the ablation of *Trpv1* gene markedly reduced the severe sight compromising responses caused by alkali burn, though it simply retards the healing of a single incision-injured cornea [41,42]. To better explain these findings, Okada et al. proposed that, in the case of a severe corneal injury, a recurrent loop linking TGFβ-1 and TRPV1 is established [36]. Consequently, the length of the pro-fibrotic signaling is extended in an activated state. This in turn results in dysregulated inflammatory reaction, myofibroblast development, fibrosis and neovascularization.

Suppression of in vivo neovascularization was demonstrated by Tomoyose et al., in a *Trpv1* gene knock out mice. After the induction of a cauterization injury at the central cornea, they showed that, in the absence of TRPV1, stromal neovascularization was inhibited, probably secondary to lower levels of both TGFβ-1 and vascular endothelial growth factor [43].

As stated before, in different tissues, TRPV1 and CB1r are co-expressed and functionally interact (i.e., colonic epithelium [44] and primary sensory neurons [45]). This strict cross-talk has been investigated in corneal tissues by Yang et al. [35,46]. In a preliminary work, they showed the two receptors not only being co-expressed on HCEC, but also being responsible for the increase in cell proliferation and migration through EGFR transactivation and MAPK/Akt-linked signaling. However, TRPV1-induced IL-6 and IL-8 release was blunted through CB1r activation [46]. Consequently, they showed that a direct agonism at CB1r is able to determine TRPV1 desensitization. The blunt increase in TRPV1-induced currents declined TAK1–JNK1 activation. As a result, promotion of effective corneal wound healing and a significant reduction in immune infiltration and consequent tissue fibrosis derived (Figure 1) [35].

Taken together these findings provide strong evidence for the involvement of TRPV1 in mediating nociception, innate immune responses and wound healing.

## 7. Peroxisome Proliferator-Activated Receptors

PPARs are members of the nuclear receptor superfamily of ligand-inducible transcription factors [47]. In mammals, there are three PPARs subtypes (PPARα, PPARδ/β and PPARγ), variably distributed among all the organs and systems [48].

Their main function is to control the expression of networks of genes involved in adipogenesis, lipid metabolism, maintenance of metabolic homeostasis and inflammation [48]. In particular, it has been widely shown that the modulation of both PPARα and PPARγ activity may regulate the inflammatory response via the modulation of the cytokines storm [49]. In fact, the activation of either PPARα or PPARγ reduces the inflammatory state by negatively interfering with the NFκB, STAT and AP-1 signaling pathways, thus inhibiting the transcription of pro-inflammatory genes such as the ones codifying per IL-2, IL-6, IL-8, TNFα and metalloproteases [49,50].

Noteworthy, both PPARα and PPARγ might be targeted by different ECBs and ECB-like compounds (i.e., AEA, palmitoylethanolamide (PEA), virodhamine, docosahexaenoyl ethanolamide (DHEA) and eicosapentaenoyl ethanolamide (EPEA)), the majority of which act as agonists on the receptor site, thus providing a net anti-inflammatory effect [51].

The expression of both PPARα and PPARγ was variably demonstrated both in conjunctiva [52], cornea [53,54] and lacrimal gland [55,56]. In a recently published paper, Mu et al. systematically described the pattern of expression of all the subtypes of the PPAR family in four ocular and periocular tissues sampled from adult rats. Specifically, they found that both PPARα and PPARγ may be detected in cornea, conjunctiva, Meibomian glands and lacrimal glands. However, among these tissues, the two aforementioned receptors resulted to be mainly expressed in the conjunctiva and lacrimal gland. In addition, a comparison of different PPARs showed that PPARγ is more expressed than PPARα in all tissues [56].

The functional role of PPAR expression in ocular and periocular tissues has been described to be linked to the regulation of the local immune-inflammatory, fibrogenic and neo-angiogenic process [57].

**Figure 2:**
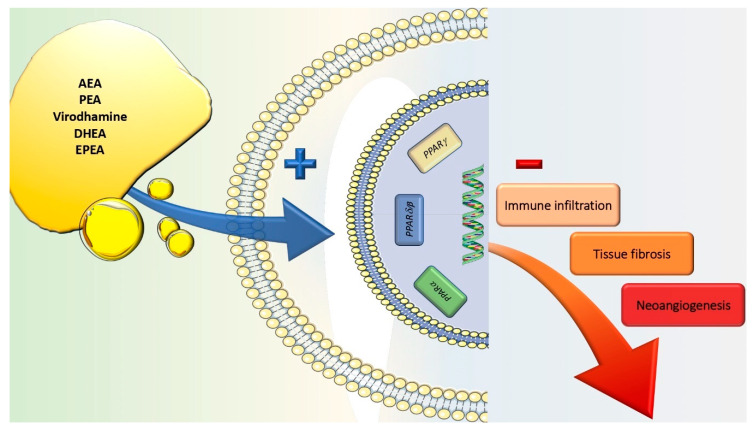
Agonism on peroxisome proliferator-activated receptor (PPAR) family members, mediated by a wide array of molecules (i.e., AEA, palmitoylethanolamide (PEA), virodhamine, DHEA and EPEA), negatively interferes with corneal tissue immune infiltration, fibrosis and neovascularization.

In a number of mouse model alkali burn injuries, the use of agonists to both PPARα and PPARγ has been shown to suppress the inflammatory state, thus reducing the aberrant fibrotic reaction and preventing neovascularization in the affected site (i.e., both conjunctiva and cornea) [58,59,60,61]. As a proof of concept, Yamanaka et al. showed that *PPARγ* gene transfer suppresses the fibrogenic reaction in cultured human subconjunctival fibroblasts as well as the injury-induced scarring of conjunctival tissue in mice [58]. This piece of evidence has been justified by the fact that PPAR activation inhibits the nuclear translocation of NFκB, thus reducing the amount of proinflammatory cytokines and preventing the infiltration of inflammatory cells via the suppression of monocyte chemoattractant protein-1 in the damaged tissue (Figure 2) [54].

In addition, the proof of a higher expression of PPARα-, β- and γ-positive cells among re-epithelialized basal cells than in normal cornea further support the hypothesis that PPARs play a major role in regulation of the healing process after corneal injuries [54].

## 8. Endogenous Cannabinoid

Cannabinoids can be classified into three groups, based on their source of production: (i) endogenous cannabinoids (endocannabinoids), (ii) natural cannabinoids (phytocannabinoids) and (iii) synthetic cannabinoids.

Endocannabinoids include lipid molecules containing long-chain polyunsaturated fatty acids, amides, esters and ethers. Among them, 2-arachidonoylglycerol and *N*-arachidonoylethanolamine or anandamide are the main representatives [51].

2-AG, an analogue of AEA containing a glycerol backbone, is a physiologically essential molecule considered as the most specific and abundant endogenous full agonist at CB1r and CB2r [21]. Notably, the tissue levels of the 2-AG are usually markedly higher than those AEA [62].

AEA belongs to the family of the *N*-acylethanolamines (NAEs). It shares many properties with THC and acts as a partial agonist of CB1r and as a weak partial agonist/antagonist of CB2r [63]. Furthermore, it should be pointed out that given the low amounts of AEA in areas with high or low density of CB receptors, it may be speculated that anandamide might activate other receptors, too [64].

Different others endogenous compounds seem to be able to modulate CB receptors. Among them, DEHA and EPEA, two derivatives of the polyunsaturated fatty acids (PUFAs) docosahexaenoic acid (DHA) and eicosapentaenoic acid (EPA), respectively, must be considered [65]. In fact, not only DEHA and EPEA are able to interact with CB receptors, but also EPA and DHA are the substrate needed for the synthesis of resolvins, lipid mediators responsible for the resolution of the inflammatory cascade under physiological conditions [65,66].

Studies have tried to evaluate the presence of ECB in the ocular surface structures. Chen et al., working with human ocular tissues (cornea, iris, ciliary body, retina and choroid) from normal and glaucomatous donors, found both 2-AG and AEA to be detectable in all the examined samples [67].

Di Zazzo et al., in an observational case-control study evaluating a cohort of patients with a new diagnosis of mucous membrane pemphigoid (MMP), detected the presence of AEA and 2-AG both in the disease and in control group [68].

As previously stated, both AEA and 2-AG were even found to be expressed in animal models of injured corneas, a notion that suggests their role in mediating local inflammatory response, pain perception and tissue wound healing [35,46].

## 9. ECS Modulation in Dry Eye Syndrome

The evidence of the strong functional role in mediating nociception, innate immune responses and wound healing has promoted the ECS as a novel therapeutic target for the management of both acute and chronic ocular surface inflammatory disorders [30,31]. Among the others, dry eye disease (DED) is one of the most investigated condition.

Dry eye is defined as a multifactorial disease of the tear fluid and ocular surface that results in symptoms of discomfort, visual disturbance and tear film instability, with potential damage to the ocular surface as per the local, dysregulated inflammatory response [69].

The first data promoting a link between the ECS modulation and DED comes from Chen et al., in 2014. In a mouse model, they proved that high levels of inflammatory cytokines, including TNFα and IL-1β, were associated with the downregulation of PPARγ expression on the ocular surface (i.e., their expression was inversely proportional) [52].

Even the modulation of TRPV1 was shown to be promising for the management of DED symptoms. Bereiter et al. demonstrated that the application of TRPV1 antagonists was able to reduce orbicularis oculi muscle activity, a marker for nocifensive behavior, in a rat model of DED [70]. These results not only promoted TRPV1 as an important mediator of nociception in DED, but also suggested a novel target for the control of ocular pain in moderate to severe cases of DED.

Based on this evidence, Di Zazzo et al. conducted a pilot, single-masked, prospective cohort study to evaluate the effect on the ocular surface of topical application of PEA eye drops (Defluxa, Medivis, Tremestieri Etneo, Catania, Italy), in patients under glaucoma treatment and suffering from DED symptoms. PEA is an endocannabinoid mimetic amide functioning as a direct agonist of PPARα and as an indirect agonist of CB1. It plays a well-known anti-inflammatory and analgesic activity. PEA eye drops treatment was shown to be effective in improving tear break up time, Schirmer test type 1 and conjunctival hyperemia without inducing any major or minor adverse event [71].

## 10. Conclusions

While the exact mechanisms underlying ocular surface inflammatory disorders are still unclear, evidence to date overwhelmingly promotes the endocannabinoid system as an important regulator and as a promising therapeutic target for the management of local immune response, wound healing and nociception [72]. However, a number of issues must be solved.

First of all, further investigation determining how the ECBS dysregulation might affect ocular surface functionality and which cellular and molecular targets should be modulated in order to restore the local homeostasis should be carried out.

Moreover, it should be noted that there are challenges in formulation of these very lipophilic compounds. Their use may, in fact, either not be able to penetrate the target organs or result in dose-dependent ocular and systemic toxicity with chronic use [73,74]. Thus, future research should explore novel cannabinoid drug combinations, appropriate routes of local delivery and evaluate both acute and chronic dosing in representative models of ocular diseases.

Finally, it must be noted that there is evidence suggesting cannabinoids have similar and, in some cases, superior efficacy and fewer side-effects as compared to traditional immunosuppressive therapeutics used in widespread clinical practice [51,75]. Clinical trials attesting that cannabinoids benefits outweigh health hazards would allow them to be legally and safely use as therapeutic devices for ocular surface diseases.
